# A Novel Procedure for the Immediate Reconstruction of Severely Resorbed Alveolar Sockets for Advanced Periodontal Disease

**DOI:** 10.1155/2017/9370693

**Published:** 2017-01-30

**Authors:** Mario Aimetti, Valeria Manavella, Luca Cricenti, Federica Romano

**Affiliations:** Department of Surgical Sciences, Periodontology Section, CIR Dental School, University of Turin, Via Nizza 230, 10126 Turin, Italy

## Abstract

*Background.* Several clinical techniques and a variety of biomaterials have been introduced over the years in an effort to overcome bone remodeling and resorption after tooth extraction. However, the predictability of these procedures in sockets with severely resorbed buccal/lingual plate due to periodontal disease is still unknown.* Case Description.* A patient with advanced periodontitis underwent extraction of upper right lateral and central incisors. The central incisor exhibited complete buccal bone plate loss and a 9 mm vertical bone deficiency on its palatal side. The alveolar sockets were filled with collagen sponge and covered with a nonresorbable high-density PTFE membrane. Primary closure was not attained and any rigid scaffold material was not used. Histologic analysis provided evidence of new bone formation. At 12 months a cone-beam computed tomographic scan revealed enough bone volume to insert two conventional dental implants in conjunction with minor horizontal bone augmentation procedures.* Clinical Implications.* This case report would seem to support the potential of the proposed reconstructive approach in changing the morphology of severely resorbed alveolar sockets, minimizing the need for advanced bone regeneration procedures during implant placement.

## 1. Introduction

Alveolar ridge resorption is a physiologic process initiated immediately after extraction leading to an average 40–60% decrease in the horizontal and vertical dimensions of the alveolar ridge during the first year [1]. Different ridge preservation techniques have been advocated to reduce the amount of bone resorption, using different bone grafting materials alone or in conjunction with barrier membranes [2]. Clinical and histologic studies reported favorable outcomes when the socket preservation therapy included sockets with intact four-wall configuration or buccal dehiscence/fenestration defects while maintaining the interdental bone peaks [3, 4].

In advanced periodontitis patients alveolar sockets often exhibit severely resorbed facial/lingual bone plate and loss of interproximal attachment level and require bone reconstruction procedures. In such patients the extraction sockets morphology often is not space maintaining. The resorption of the interproximal bone peaks as well as the inadequate soft tissue volume makes it difficult to ensure graft material stability and to obtain primary wound closure. This may jeopardize the hard tissue volume reconstruction. Therefore, the aim of the present case report is to describe the clinical and histologic outcomes of a novel procedure for the immediate reconstruction of severely resorbed alveolar sockets for advanced periodontal disease.

## 2. Case Presentation

### 2.1. Patient and Alveolar Socket Reconstruction

**Figure 1:**
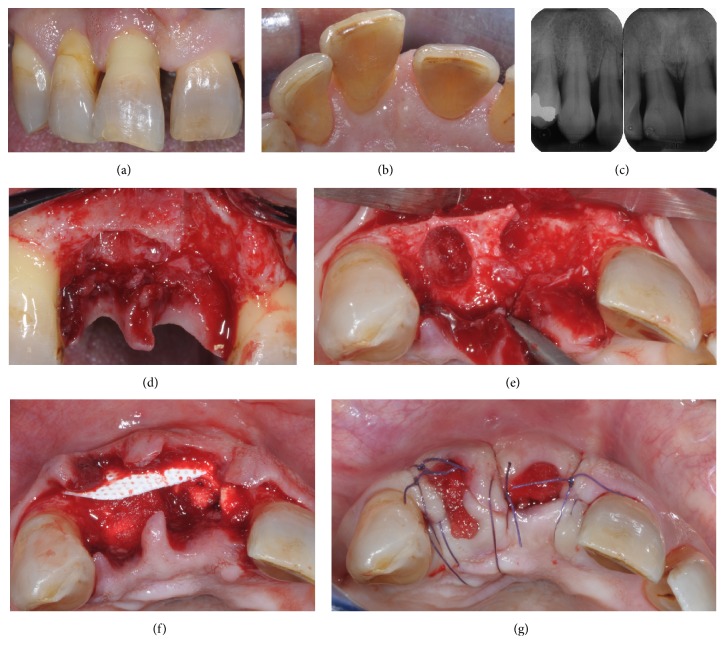
Maxillary right central and lateral incisors before extraction (a). Note extrusion of central and lateral incisors, migration of central incisor, and persistent inflammation (b). Preoperative radiographs showing severe interdental bone loss and widening of the residual periodontal ligament space as a consequence of the occlusal trauma (c). Intraoperative view following teeth removal (d). Occlusal view showing vertical bone resorption and partial nonspace maintaining defect at central incisor (e). After placement of a nonresorbable d-PTFE membrane to replace the missing buccal bony wall, the gap between the membrane and the residual palatal wall was filled with the collagen sponge (f). Flaps were repositioned and the membrane was left partially exposed and protected with the collagen sponge (g).

A healthy, nonsmoker 48-year-old male presented with the chief complaint of gingival bleeding. The comprehensive periodontal examination and full-mouth periapical radiographs revealed a generalized severe chronic periodontitis. After etiological periodontal treatment was completed, the right maxillary incisors exhibited persistent suppuration, tooth number 7 had mobility grade II/III, and tooth number 8 had mobility grade III. Both teeth were extruded and tooth number 8 exhibited pathologic migration with a midline diastema of 2-3 mm (Figures 1(a)–1(c)). A combined approach consisting of regenerative periodontal surgery and orthodontic therapy was proposed to the patient as ideal treatment plan to try to retain compromised teeth. Given the unwillingness of the patient to undergo an orthodontic treatment, the achievement of clinical outcomes compatible with a good long-term prognosis was considered unpredictable. The patient was made aware of the consequences associated with his decision and of risks of retaining severely compromised teeth and consented to an implant-supported fixed rehabilitation. The extractions of tooth number 7 and tooth number 8 were scheduled together with the alveolar ridge reconstruction procedure. The patient signed informed consent.

At the time of tooth extraction, mucoperiosteal buccal and palatal flaps were reflected without vertical releasing incisions. The reflection of the palatal flap was limited to the most coronal portion of the alveolar crest. Minimally traumatic extractions were performed. The analysis of the alveolar sockets morphology confirmed the complete buccal bone wall deficiency at tooth number 8 and a vertical bone height deficiency of about 9 mm on its palatal side (Figures 1(d)-1(e)). The sockets were thoroughly debrided to remove the granulation tissue and to enhance vascular supply by opening the trabecular bony spaces.

A 25 × 30 mm textured high-density polytetrafluoroethylene (d-PTFE) nonresorbable membrane (Cytoplast GBR-200 singles, Osteogenics Biomedical, Lubbock, TX) was trimmed and placed under the buccal flap to replace completely the missing bony wall. A commercially available bioabsorbable sponge of equine lyophilized type I collagen (Gingistat, GABA, Italy) was used to fill the gap between the membrane and the residual palatal wall (Figure 1(f)). Afterwards, the membrane was tucked under the palatal flap taking care to keep the edge of the membrane a minimum of 1 mm away from the adjacent roots. The flaps were repositioned without periosteal incisions and secured with vertical and horizontal mattress sutures (Vicryl, Ethicon, Johnson & Johnosn, Sint-Stevens-Woluwe, Belgium). Intentional primary closure was not attempted and the membrane was left partially exposed and protected by the collagen sponge (Figure 1(g)). Postoperative care comprised 0.12% chlorhexidine mouthrinse three times daily for 2 weeks, systemic amoxicillin (1 g twice a day) for 6 days, and ibuprofen (600 mg twice a day) for 5 days. Sutures were removed after 14 days. A removable transparent acetate mask replacing upper incisors was used as provisional restoration to avoid any pressure on the underlying tissue.

**Figure 2:**
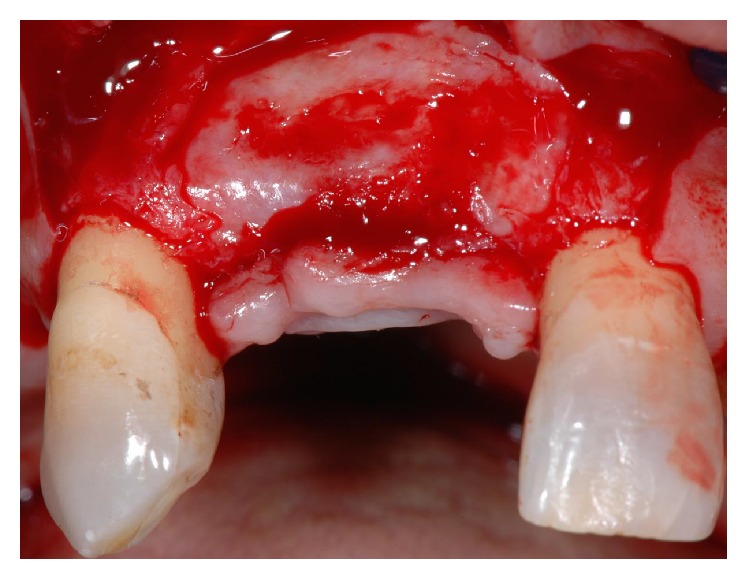
At 4 months the extraction sockets were completely filled by uniform radiodense bone tissue. Note the ridge morphology mimicking the space created beneath the membrane.

At week 6 the membrane was gently removed from the tissue bed without flap elevation by means of a tissue forcep. Beneath the membrane a dense and highly vascularized connective tissue was detected. Three months later a corrective plastic surgery was carried out at the area of ridge reconstruction to increase soft tissue volume and so to obtain a more natural prosthetic emergence profile. After flap elevation, bone formation was observed with a ridge morphology mimicking the space created beneath the membrane (Figure 2). Before placing the palatal connective tissue over the recipient site, two approximately 5 mm long sample cores of newly formed tissue were collected for histologic examination from the central region of the former tooth sockets by using the bone chip extractor with a 2.9 mm internal diameter (9126 Komet srl, Italy). Native bone was not included. Specimens were marked to identify the coronal and apical ends. Paraffin blocks were sectioned in the apicocoronal plane to obtain 4 *μ*m thick sections and stained with hematoxylin-eosin. The sections were evaluated by light microscopy to quantify bone content. Computer-assisted histomorphometric measurements of the newly formed bone were obtained using image analysis software (QWin, Leica Microsystems, Buffalo Grove, IL) on four to six fields for each section.

### 2.2. Clinical and Histologic Outcomes

**Figure 3:**
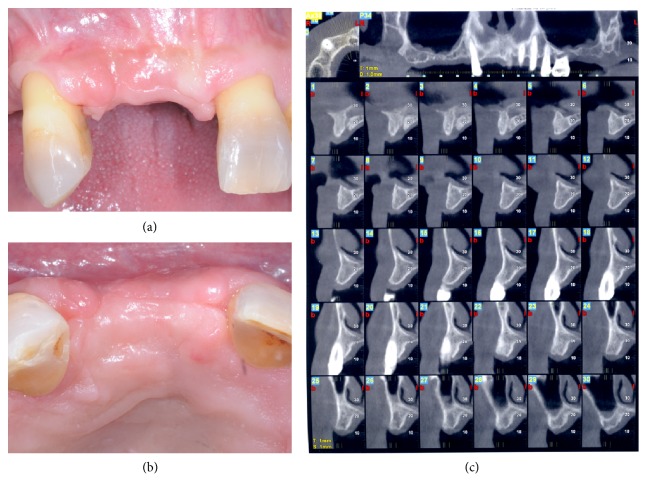
One year after the ridge reconstruction procedure ridge regeneration was achieved. Buccal view (a); occlusal view (b); cone-beam computed tomographic scan (c).

During the 12 months of observation no signs of infection were noted. At 12 months postoperatively a cone-beam computed tomographic scan (CT) was taken in order to plan the implant-supported fixed prosthesis. Resolution of the alveolar bone deficiencies on tooth number 8 and limiting of the physiologic ridge reduction were attained (Figure 3). The edentulous ridge had a vertical dimension between 13 mm and 14 mm and a horizontal dimension of 5 mm on its proximal sides and of 4.3 mm on the center of the former lateral incisor socket below the most coronal part of the crest. The final ridge dimensions allowed for insertion of two conventional dental implants in conjunction with a minor horizontal bone augmentation procedure to increase the buccopalatal crestal bone width. Unfortunately, the patient refused implant placement for unexpected incoming financial reasons and consented to replace the missing teeth with a conventional removable rehabilitation.

**Figure 4:**
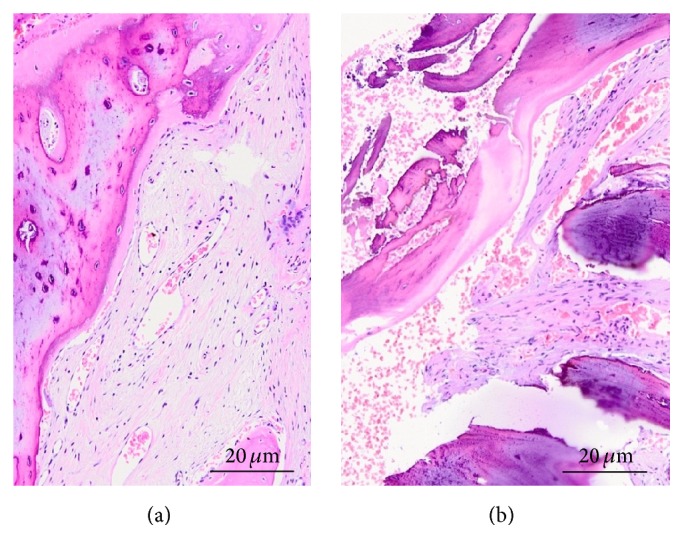
Bone biopsy illustrating socket healing 4 months after the ridge reconstruction procedure. Hematoxylin and eosin staining. Total magnification: ×100 ((a), central incisor; (b), lateral incisor).

Histologic analysis revealed new bone formation through the entire length of the specimens without signs of inflammation. The newly formed bone was well structured with intense osteoblastic activity and it consisted of 100% living trabecular bone. The connective tissue was free of inflammation and well vascularized in all the examined sections. The overall mean percentage of newly formed bone was 49.3%*  *±*  *4.7% and was composed mostly by lamellar bone (33.2%*  *±*  *3.6%) (Figure 4). In the crestal region the mineralized fraction amounted to 47.1%*  *±*  *3.9%, and in the most apical part of the specimens it was 51.0%*  *±*  *6.2%.

## 3. Discussion

This case report describes a novel treatment approach in the immediate alveolar reconstruction of severely resorbed extraction sites due to periodontal disease. In the literature several data are available on the use of the guided bone regeneration (GBR) procedures to reconstruct alveolar ridge defects prior to or in conjunction with osseointegrated implants placement [5]. However, GBR techniques, in presence of severe atrophy, are complex and technically demanding. The most important factor limiting the amount of new bone formation is the early membrane exposure and infection.

In this case report, two maxillary incisors were atraumatically extracted, and the sockets were filled with a collagen sponge and covered with a nonresorbable d-PTFE membrane. Primary closure was not attained and any rigid scaffold material was not used. At 12 months the radiographic bone volume was enough to insert two implants in conjunction with a minor horizontal bone augmentation procedure. Histologically, the mean percentage of newly formed bone was 49.3%*  *±*  *4.7%. These findings are encouraging when considering the unfavorable anatomy of the alveolar socket on tooth number 8 which displayed complete loss of the buccal bone wall and a 9 mm vertical bone deficiency on its palatal aspect. It is reasonable to assume that such residual alveolar crest would undergo more contraction compared with a 4-wall extraction socket defect when is left to heal spontaneously. Tooth extraction without the use of grafting materials leads to percentage of horizontal dimensional changes of 29–63% after 6-7 months. Such bone remodeling is further complicated if the buccal bone wall is lost as a result of inflammatory processes or the extraction itself. Lee et al. observed a mean decrease of about 60% in the horizontal dimensions in buccal-bone-deficient alveolar sockets during the first 8 weeks of healing [6].

The present results may be explained by the combined use of d-PTFE membrane and type I collagen materials. The optimal antimicrobial effects and durability of d-PTFE membrane protect the clot from both mechanical and chemical stress. Due to its submicron porosity (<0.3 *μ*m) preventing or minimizing bacterial penetration, the d-PTFE membrane may provide a reasonably microbe-free environment to the underlying collagen material, thereby facilitating its cell adhesive properties [7]. In the present report an equine type I collagen sponge was used as filling material to promote and stabilize clot formation in the early stages of healing and to avoid any interference in the later bone regeneration stages due to the inflammatory response when the filling material starts to degrade. Type I collagen has shown proangiogenic qualities and acceleration of ingrowth, proliferation, and maturation of endothelial cells that encourage physiological bone regeneration [8].

The rationale of the proposed modification of the conventional socket grafting approach relies on the findings by Serino et al. [9]. They used a polylactide-polyglycolide sponge as space filler in alveolar postextraction sockets without primary closure of the surgical wound. The histologic analysis at 6-month follow-up showed mature and well-structured bone with no residual particles of the grafted material. In this regard, it should be noted that studies in humans using demineralized freeze-dried bone allograft or deproteinized natural bovine bone have shown variable amount of particles of the grafted material in the alveolar sockets 4–9 months following their insertion [10–12]. Histologic examinations demonstrated that such biomaterials retard the extraction socket healing when compared to the naturally healing control [13]. Nevertheless, a recent systematic review reported less mid-buccal height loss in 4-wall intact sites grafted with a xenograft but comparable decrease in mid-lingual bone height and in buccolingual width when compared to alloplastic materials during socket preservation procedures [14].

Another aspect to be considered is the lack of requirement of primary closure over the socket. Because of d-PTFE membrane small porosity and minimal inflammation when exposed to the oral environment soft tissue coverage is not needed [15]. This is a significant advantage over e-PTFE and resorbable membranes in ridge augmentation application. The use of d-PTFE membranes without primary closure allows the clinician to preserve the existing keratinized tissue width and to achieve the regeneration of keratinized tissue over the extraction site [15].

## 4. Conclusion

Although only a single patient was treated using the present technique, clinical and histologic results are encouraging. They do indicate that this postextraction reconstructive procedure, even with open healing, may promote bone formation, improve ridge shape and dimension in severely resorbed alveolar sockets, and simplify later treatment procedures during implant placement. In addition, it can be easily managed without requiring high surgical skill. Further studies with higher patient number and long-term follow-ups are needed to validate this procedure.
